# High-speed raster-scanning synchrotron serial microcrystallography with a high-precision piezo-scanner

**DOI:** 10.1107/S1600577518010354

**Published:** 2018-08-23

**Authors:** Yuan Gao, Weihe Xu, Wuxian Shi, Alexei Soares, Jean Jakoncic, Stuart Myers, Bruno Martins, John Skinner, Qun Liu, Herbert Bernstein, Sean McSweeney, Evgeny Nazaretski, Martin R. Fuchs

**Affiliations:** a Photon Sciences, Brookhaven National Laboratory, Upton, NY 11973, USA; b Case Center for Synchrotron Biosciences, Case Western Reserve University, OH 44106, USA; c School of Chemistry and Materials Science, Rochester Institute of Technology, NY 14623, USA

**Keywords:** macromolecular crystallography, nano-positioning, serial crystallography, raster-scanning, high-precision goniometer

## Abstract

The Frontier Microfocus Macromolecular Crystallography (FMX) beamline at the National Synchrotron Light Source II with its 1 µm beam size and photon flux of 3 × 10^12^ photons s^−1^ at a photon energy of 12.66 keV has reached unprecedented dose rates for a structural biology beamline. The high dose rate presents a great advantage for serial microcrystallography in cutting measurement time from hours to minutes. To provide the instrumentation basis for such measurements at the full flux of the FMX beamline, a high-speed, high-precision goniometer based on a unique *XYZ* piezo positioner has been designed and constructed. The piezo-based goniometer is able to achieve sub-100 nm raster-scanning precision at over 10 grid-linepairs s^−1^ frequency for fly scans of a 200 µm-wide raster. The performance of the scanner in both laboratory and serial crystallography measurements up to the maximum frame rate of 750 Hz of the Eiger 16M’s 4M region-of-interest mode has been verified in this work. This unprecedented experimental speed significantly reduces serial-crystallography data collection time at synchrotrons, allowing utilization of the full brightness of the emerging synchrotron radiation facilities.

## Abstract 2

The design and first measurements with a high-speed piezo-positioner-based goniometer for raster-scanning serial macromolecular micro-focus crystallography at synchrotron storage rings are presented.

## 1. Introduction   

In macromolecular crystallography (MX), serial crystallography has been the method of choice to overcome limitations in sample dose life under X-ray irradiation. Before the advent of cryocrystallography (Hope, 1988 ▸; Henderson, 1990 ▸), the merging of partial datasets from multiple crystals was used to deal with the limited dose life (Kendrew *et al.*, 1960 ▸) and continues to be used in room-temperature crystallography for hard-to-freeze crystals, such as virus crystals (Fry *et al.*, 1999 ▸; Axford *et al.*, 2012 ▸; Rossmann, 2015 ▸).

At free-electron lasers, the ‘diffraction before destruction’ approach (Chapman *et al.*, 2014 ▸) has crystals surviving only a single exposure and this therefore dictates serial crystal delivery; the crystal sample is destroyed during the measurement and must be replaced by a new sample for further diffraction measurements. With the latest generation of high-brilliance storage-ring sources, beamlines are being constructed that enable XFEL-like serial crystallography to be performed at storage-ring facilities (Yamamoto *et al.*, 2017 ▸). The appeal of these instruments is that they enable crystallographic data collection from microcrystals that would previously have been too small to yield useable diffraction data.

In a pioneering experiment at beamline P14 at the PETRA3 electron storage ring, the methods from FEL serial-femto-second crystallography and synchrotron-based MX were successfully combined (Gati *et al.*, 2014 ▸). In these measurements, a 3 Å-resolution structure was obtained by rastering a frozen slurry of microcrystals of cathepsin B across a microfocus beam and merging the diffraction images from hundreds of microcrystals to make a complete crystallographic dataset. Experiments at the ESRF beamline ID13 have extended this scanning serial method to room-temperature data collection by containing the microcrystals in an Si_3_N_4_ membrane sandwich to avoid the drying of the samples while limiting background scattering (Coquelle *et al.*, 2015 ▸).

These scanning serial crystallography methods dramatically reduce sample consumption when compared with techniques employing liquid jets (Weierstall, 2014 ▸) or flow capillaries (Stellato *et al.*, 2014 ▸). For crystals not grown in lipidic cubic-phase media, they avoid the need to introduce the crystals into a viscous medium for the high-viscosity extrusion injectors (Nogly *et al.*, 2015 ▸) and the associated background scattering. The acquisition of several sequential rotation exposures in the cryo-rastering experiment simplifies data treatment and obviates the need for Monte Carlo averaging of still images and a further reduction in the amount of crystals required to record a complete dataset. The increased number of reflections recorded in a rotation image and the known partialities of the recorded reflections aid indexing and data integration (Hasegawa *et al.*, 2017 ▸; Gati *et al.*, 2014 ▸). Even in cases where larger crystals of a compound are available, the advantages of microcrystallography may make this method appealing by avoiding averaging over crystal inhomogeneities, reduced mosaicity (Bowler *et al.*, 2010 ▸), reduced radiation damage attributed to photoelectron escape in the case of micrometre-sized crystals (Sanishvili *et al.*, 2011 ▸) and increased uniformity during ligand diffusion (Stagno *et al.*, 2017 ▸).

Recently constructed synchrotron storage rings achieve ever lower horizontal electron-beam emittances from 1.2 nm rad for PETRA III (Wanzenberg *et al.*, 2015 ▸), over 0.9 nm rad for NSLS-II (Willeke, 2015 ▸) to 0.3 nm rad for MAX IV (Eriksson *et al.*, 2016 ▸), permitting the design of MX beamlines that deliver microfocus beams with high flux densities. Specifically, the FMX beamline in sector 17-ID-2 of NSLS-II currently delivers 3 × 10^12^ photons s^−1^ into a 1 µm (V) × 1.5 µm (H) beam focus (Fuchs *et al.*, 2016 ▸). This flux density surpasses the brightest current MX beamlines by up to two orders of magnitude. For the unattenuated beam at FMX at 12.66 keV, the estimated maximum dose reaches the 30 MGy Garman limit (Owen *et al.*, 2006 ▸) in as short as 40 ms, while considering a protein crystal matched to the beam size.

A common factor to raster-scanning serial crystallography approaches at storage rings reported to date (Gati *et al.*, 2014 ▸; Coquelle *et al.*, 2015 ▸) are data collection times in the hours range as a result of available dose rates and goniometer speeds. Fixed-target supports with crystals loaded onto regularly spaced grids enable raster scans with significantly increased hit rates close to 50% (Owen *et al.*, 2017 ▸) and up to >90% (Roedig *et al.*, 2017 ▸), and thereby achieve data collection times under 10 min.

In addition to fixed rasters, the new goniometer at FMX supports fly-scanning mode without stopping the goniometer or closing the shutter. This comes at much higher collection speeds that overcome the lower hit rates. In addition, crystals can be loaded onto unstructured membranes to facilitate sample-holder loading without boundary conditions on the crystal shapes. With the new goniometer’s scanning speed, we demonstrated data collection times of a few minutes, with the actual open-shutter time under 20 s. By bringing raster-scanning serial crystallography collection times from the hours range into the time range of a classical single-crystal data collection, these experiments become an efficient new way of collecting diffraction datasets from previously intractable crystals. In addition, MX at such extreme dose rates promises two further key advantages: an increase in time resolution and the potential to outrun radiation-damage effects (Warkentin *et al.*, 2017 ▸, 2013 ▸; Owen *et al.*, 2012 ▸).

The focus of our present work is to enable rapid-scanning serial macromolecular crystallography experiments under cryo-conditions and room temperature at the full flux of the fully focused FMX beamline. The system we developed has the potential for application well beyond the protein crystallography community; X-ray imaging and microscopy will benefit from this development. Full-field transmission X-ray microscopes require fast and accurate rotation to acquire three-dimensional tomograms (Wang *et al.*, 2012 ▸; Hwu *et al.*, 2013 ▸). Nanometre-scale resolution scanning fluorescence imaging systems also require accurate rotation accompanied by a fast fly-scanning capability (Naza­retski *et al.*, 2015 ▸, 2017 ▸; Holler *et al.*, 2014 ▸). The capability of the system we developed to perform infinite rotation makes it even more appealing for *in situ*, *in operando* measurements performed during transmission, fluorescence, diffraction or ptychography experiments (Liu *et al.*, 2016 ▸; Nazaretski *et al.*, 2013 ▸, 2014 ▸; Shapiro *et al.*, 2014 ▸).

## 2. Materials and methods   

### 2.1. Instrument design   

To realize the promise of experiments at the FMX beamline’s highest achievable dose rates, sample positioning and movement requires uncompromising design decisions for the goniometer spindle and experimental station. Here we present the newly developed FastForward Goniometer, a design that is not limited by the requirements of multi-axis macromolecular crystallography goniometers.

The experimental boundary conditions inform the requirements for the goniometry to be developed. The 750 Hz maximal detector frame rate of the FMX’s Eiger 16M detector in a 4M region of interest (ROI) coincides well with a 3% fraction of the estimated 40 ms half-life. For the goniometer translations, a raster pitch with a 1 µm beam size yields the scanning-speed requirement of 1 mm s^−1^. This is a lower bound when considering overlapping beam tails, as well as potential flux upgrades to the beamline, *e.g.* by increasing the monochromator bandwidth. The chosen design can achieve linear scanning speeds of >10 mm s^−1^. The main challenge for achieving fast rastering performance are the resulting high line-scanning frequencies. At 1 mm s^−1^, for a 100 µm raster size, the associated 10 Hz scanning frequency will lead to unacceptable mechanical resonances in typical MX goniometer systems. Here we present the design and demonstrate the performance of a piezo positioner-based goniometer with a 200 µm × 200 µm × 200 µm travel range and 25 nm bi-directional repeatability, which can raster the full scan area in 20 s with a 1 µm step size. Combined with a 4 mm travel range coarse piezo-walk centering stage, it is applicable to both classical cryo-crystallography and high-speed raster-scanning serial crystallography.

**Figure 1:**
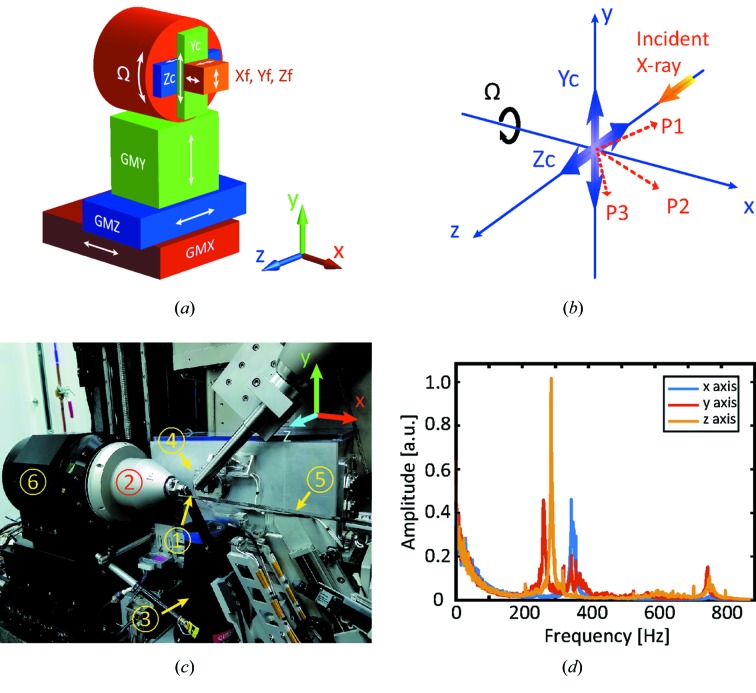
Piezo scanner in the FMX endstation. (*a*) Schematic of the system we developed. *X*f, *Y*f and *Z*f are the fine-scanning directions with a 200 µm travel range in each direction. *Y*c and *Z*c are the coarse motion directions. Ω is the rotation around the *X*-axis. GM*X*, GM*Y* and GM*Z* are the motion directions of the *XYZ* stage assembly. (*b*) Schematic of the piezo scanner. *P*1, *P*2 and *P*3 are the motion directions of three shear piezo actuators of the NanoCube scanner. *Y*c and *Z*c are the motions of the NEXLINE coarse stages. Both fine and coarse stages are mounted on top of an air-bearing rotational stage, Ω. (*c*) Photograph of the system we developed installed at the FMX endstation, key components are enumerated: (1) cryogenic sample loaded on a SPINE cap, (2) piezo scanner, (3) microscope, (4) collimator, (5) beam stopper, (6) Nelson Air rotary stage. (*d*) Resonance frequencies of the piezo scanning system we developed.

The FastForward Goniometer system was developed in collaboration with Physik Instrumente and utilized modified NEXLINE and NanoCube stages as subparts. To achieve the desired scanning speed and precision, as well as to fit the system into the limited space of the FMX endstation, the scanner utilizes three piezo actuators arranged diagonally with respect to the laboratory coordinate system (see Fig. 1 ▸ *b*). When compared with a stack of individual linear-motion stages, this diagonal arrangement yields a much smaller form factor of the entire assembly. When arranged diagonally, the stiffness of the stage we developed, which determines the highest scanning frequency it can achieve, is much higher than that of a simple motion stack. Moreover, the combined motion of all three piezo actuators allows a performance of not only translational but also rotational motion around an arbitrarily selected pivot point. Owing to the fact that shear piezo actuators are utilized in the scanner we developed, its scanning range is limited to 200 µm in all three directions. To overcome the travel-range limitation, the fine-scanning stage is mounted on top of coarse linear actuators with a travel range of 4 mm in the *Y* and *Z* directions, respectively [see Fig. 1( ▸ *a*) for a schematic of the setup].

Fig. 1( ▸ *c*) demonstrates the system that we developed installed at the FMX endstation, all key components enumerated. The piezo scanner system is mounted on a horizontal high-precision air-bearing rotational axis (Nelson Air SP150), which in turn resides on top of an *XYZ* stage assembly. This scanning system allows for infinite rotation around the *x* axis, *i.e.* Ω in Fig. 1( ▸ *a*); therefore all actuators and encoders of the scanner are connected through a sliding cable connector (the so-called slip ring) which is a part of the Nelson Air rotary stage. Upon completion, the manipulation system we developed was first characterized in the metrology laboratory of NSLS-II and then tested at the FMX endstation.

### 2.2. Sample preparation   

Rod-shaped bovine trypsin crystals and proteinase K microcrystals were chosen as the test crystals for our experiments. Trypsin crystals were grown on siliconized glass cover slips (hanging drop) by equilibrating 15 mg ml^−1^ trypsin (Sigma–Aldrich; T1426) plus 5 mg ml^−1^ benzamidine and 10 m*M* calcium chloride in 20 m*M* HEPES (pH 7.0) with 3.75% PEG 3350 and 5% glycerol over a reservoir containing 15% PEG 3350 and 20% glycerol. Large trypsin crystals formed after ∼7 days, surrounded by thin trypsin needles. Proteinase K crystals were grown on siliconized glass cover slips (hanging drop) by equilibrating 25 mg ml^−1^ proteinase K (Worthington Biochemical; LS004224) in 40 m*M* calcium chloride, 400 m*M* sodium nitrate and 50 m*M* BisTris (pH 6.5) over a reservoir containing 160 m*M* calcium chloride, 1.6 *M* sodium nitrate and 200 m*M* BisTris (pH 6.5). To generate 5 µm-sized microcrystals, each crystallization drop was allowed to air dry for ∼5 min, until an opaque layer of sub-micrometre crystals formed, and then the cover slip was mounted over the reservoir. The surface layer of crystals continued to grow overnight to a final size of 5 µm and were cryo-protected by 30% ethyl­ene glycol prior to harvesting.

The sample crystals were harvested from the mother liquor using loops or meshes on SPINE caps. The samples were then flash-cooled and stored in liquid nitro­gen until mounted on the goniometer using a permanent magnet. In a final system update, a smart magnet (Arinax), which can detect the presence of mounted specimens, will replace the permanent magnet to enable automatic sample changing. A nitro­gen coolant gas stream (Cryostream 800 system, Oxford Cryosystems) provided a sample temperature of 100 K.

## 3. Results   

### 3.1. Instrument characterization   

Prior to deployment at the beamline, the system that we developed was thoroughly characterized at the metrology laboratory of NSLS-II and basic performance characteristics were inferred.

#### 3.1.1. Stiffness   

The stiffness of a system, usually characterized by its fundamental resonance frequencies, determines the highest scanning frequency the system can achieve. We utilized a three-axis laser interferometer (Attocube FPS3010) to measure displacements of the scanning stage in the *X*, *Y* and *Z* directions. The recorded time domain signal was Fourier transformed and the frequency domain spectrum was obtained, see Fig. 1( ▸ *d*). The fundamental resonance frequencies in the *X*, *Y* and *Z* directions correspond to 380 Hz, 275 Hz and 290 Hz, respectively, which allows us to achieve scanning frequencies exceeding 100 Hz.

#### 3.1.2. Resolution and repeatability   

**Figure 2:**
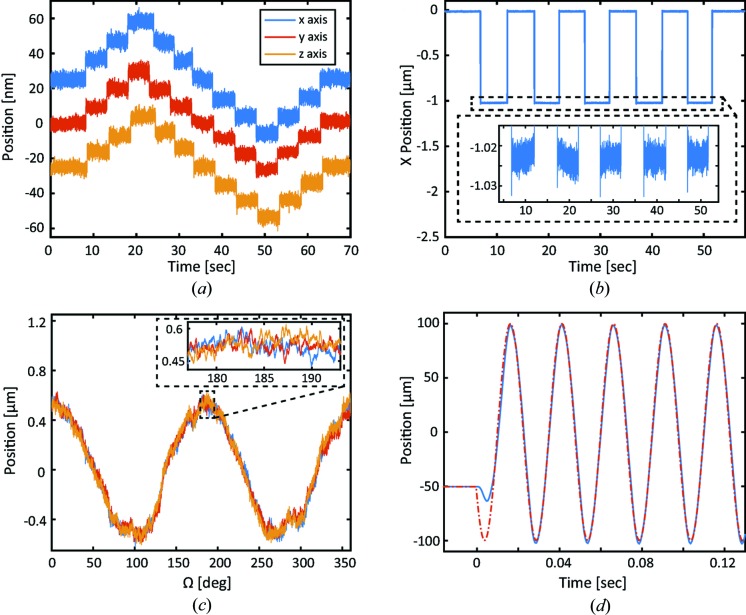
Laboratory characterization of the piezo scanner. (*a*) Resolution measurements, 10 nm steps in the *X*-, *Y*- and *Z*-directions directly measured using interferometry. (*b*) Repeatability measurements, 1 µm steps in the *X*-direction performed with the fine piezo stage. (*c*) Radial runout error measured using capacitive displacement sensors. (*d*) Scanning trajectory measured in the *x*-direction at a 40 Hz scanning frequency (blue solid line). The red dashed line demonstrates the selected trajectory.

The resolution and repeatability of the piezo scanner were directly measured using the same three-axis interferometer. In order to improve the background noise and stability of the measurements, the piezo scanner was mounted on a home-built aluminium support structure installed on a pneumatically suspended optical table. To minimize the influence of acoustic noise, the system we developed was kept inside a double-wall sound-absorbing enclosure. As shown in Fig. 2 ▸(*a*), steps of 10 nm in all three directions are clearly resolved. The noise floor of approximately 10 nm in all three directions was mainly determined by environmental contributions. When fully assembled and powered through a slip-ring, the background noise increased to 25 nm because of the friction-based mechanical connection inside the slip-ring.

Similar measurements over a travel distance of 1 µm were also performed to determine the repeatability of the motion. Fig. 2 ▸(*b*) demonstrates a 1 µm repetitive motion in the *X*-direction, with a repeatability better than 5 nm. A 2 nm slow drift observed during the 10 s wait time at each step (see inset in Fig. 2 ▸ *b*) was caused by thermal fluctuation. For larger steps (in excess of 100 µm) the measured bidirectional repeatability was determined to be better than 25 nm. All three axes demonstrated similar performances. For coarse motions, repeatability was determined to be 135 nm and 77 nm for the *y* axis and *z* axis, respectively, for a travel range >2 mm.

In the endstation, the background-noise measurement using external interferometry was not feasible with the space constraints. Here, readings from the piezo stages’ internal encoders were utilized to estimate the noise level, which was 150 nm peak-to-peak.

#### 3.1.3. Sphere of confusion of a rotary stage   

Apart from resolution and repeatability, the sphere of confusion of a rotary stage plays an important role in defining the accurate position of a sample during experiments. We characterized rotational errors by measuring the trajectory of a diamond-turned reference cylinder installed on top of a rotary stage during rotational motions. The cylinder is 30 mm tall and 25 mm in diameter with the surface roughness better than 0.2 nm and circularity better than 30 nm. The setup, reference cylinder and measurement procedure are similar to those described by Xu *et al.* (2014 ▸) and Kim *et al.* (2013 ▸). Fig. 2 ▸(*c*) demonstrates rotational errors obtained during the measurements. The total runout error is 1 µm peak-to-peak with a 150 nm asynchronous error (see inset in Fig. 2 ▸ *c*). The synchronous (repeatable) error will be incorporated into the control algorithms and compensated for by a piezo-scanner during scanning measurements. The axial runout was determined to be 40 nm peak-to-peak.

#### 3.1.4. Accuracy of high-speed scanning   

The trajectory of a high-speed scan was measured by external interferometry. A typical two-dimensional scan involves fast motion in the *X*-direction and slow motion in the *Y*-direction. Fig. 2 ▸(*d*) shows a comparison between the targeted position and the actual position measured by the interferometer at a 40 Hz scanning frequency. The scanning range was maximized and reached ±100 µm in the *X*-direction. The bidirectional repeatability was determined to be frequency dependent, varying from <100 nm at 20 Hz scanning frequency and reaching the maximum of 300 nm at 40 Hz scanning frequency and above.

### 3.2. Beamline experiments   

Following laboratory characterization, the piezo scanner was installed at the FMX endstation for crystallographic data collection. During the measurements, diffraction data were recorded by an Eiger 16M detector, which can achieve up to a 133 Hz full frame rate and 750 Hz frame rate in a 4M ROI.

For evaluation of the performance of the goniometer and scanner system in crystallographic experiments, the most common combined-motion data collection strategies were characterized.

#### 3.2.1. Vector scan   

In order to reveal potential systematic errors in the hardware and/or software, the piezo scanner was first tested by performing vector scans along rod-shaped crystals of bovine trypsin with average dimensions of 25 µm × 25 µm × 200 µm. In vector scans, the goniometer performs a combined motion of the rotation axis and the *X*f, *Y*f and *Z*f motions to translate the crystal across the beam in a continuous exposure.

The experiment was conducted at a photon energy of 12.66 keV, a flux of 8.8 × 10^10^ photons s^−1^, a beam size of 2 µm (V) × 4 µm (H) FWHM and the detector operating in full-frame mode. After centering the selected crystal with respect to the X-ray beam using the on-axis microscope, diffraction images were collected in a continuous fly scan over a rotation range of 180° with 0.1° per frame at an exposure time of 50 ms. During the rotation, the sample was translated 0.5 µm deg^−1^ to spatially distribute the radiation dose over a 90 µm range. The average dose in the exposed region was calculated to be 3.6 MGy *via* *RADDOSE-3D* (Zeldin *et al.*, 2013 ▸). Demonstrating the smoothness of the goniometer movement, the scale factor of the processed dataset varies between 0.47 and 0.52 (Fig. S1 of the supporting information).

The diffraction patterns from the vector scan were indexed and integrated using the fast_dp processing pipeline (Winter & McAuley, 2011 ▸; Kabsch, 2010 ▸; Grosse-Kunstleve *et al.*, 2002 ▸; Winn *et al.*, 2011 ▸). The quality and internal consistency of the data were judged on the basis of standard 〈*I*/σ(*I*)〉 statistics, as well as on the basis of CC(1/2), the correlation percentage between intensities from random half-datasets (Karplus & Diederichs, 2012 ▸). The diffraction data were processed to a resolution of 1.52 Å, with an overall *R*_merge_ of 0.041 and a CC(1/2) of 99.9% (Table 1 ▸). The 〈*I*/σ(*I*)〉 in the highest-resolution shell was 1.6. Structure refinement was conducted *via* *PHENIX* (Adams *et al.*, 2010 ▸) with an initial model from PDB entry 3t26. The resultant *R*_free_ = 0.189.

#### 3.2.2. Raster scan   

For high-speed raster-scanning serial crystallography data collection, stiff MiTeGen polyimide sample supports were utilized for stability during scanning and to maximize the scanning speed. We had greatest success with efficient crystal loading and low background scatter using MicroLoop and customized MicroWell pins (Guo *et al.*, 2018 ▸) from MiTeGen. Diffraction experiments were conducted at a photon energy of 12.66 keV, beam size of 1 µm (V) × 2 µm (H) FWHM, maximum unattenuated flux of 1.8 × 10^12^ photons s^−1^ at a ring current of 300 mA at the time of the experiment and with the detector in 4M ROI mode to achieve the required frame rates.

**Figure 3:**
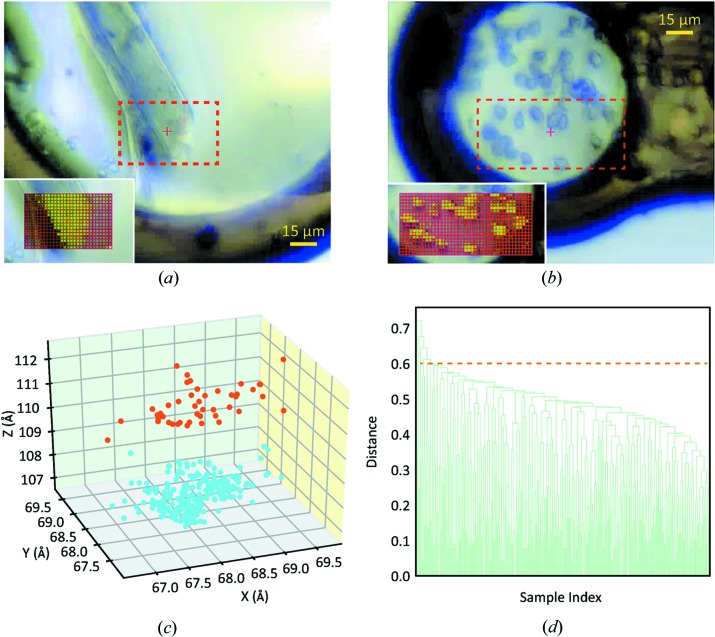
Crystallography data collection and processing at FMX. Raster scans of (*a*) a bovine trypsin rod-shaped crystal and (*b*) 5–10 µm-sized proteinase K crystals loaded on MiTeGen loops. The raster-scan areas are shown as red-dashed rectangles; the corresponding heat maps acquired after the raster scans are shown in the insets. The grid coloring is the diffraction pattern spot count. (*c*) Clustering of the serial crystallography partial datasets based on unit-cell size. The data shown were acquired from raster scans of proteinase K microcrystals with a 200 Hz detector frame rate. Of these 279 partial datasets, 234 (blue dots) were indexed to *c* = 106.58 ± 0.41 Å, with 99.6% completeness (Table 1 ▸). The remaining 45 partial datasets (orange dots) with *c* = 110.06 ± 0.84 Å did not yield a complete dataset and were excluded from refinement. The unit-cell size was converted to Cartesian coordinates for better visualization. The data were clustered into two groups using a *K*-mean clustering algorithm. (*d*) Hierarchical cluster-analysis dendrogram for the proteinase K partial datasets acquired at a 750 Hz detector frame rate. The cutoff limit was set to 0.6 (dashed line).

Raster scans of the trypsin crystals with detector frame rates of up to 750 Hz were conducted in a test experiment to calibrate the synchronization between the piezo scanner and detector. After the raster scan, *Spotfinder* (Sauter, 2010 ▸, 2011 ▸) was utilized to evaluate the quality of each diffraction pattern. A heat map, based on the spot count determined by *Spotfinder*, demonstrated the location and the morphology of the protein crystal. The synchronization of spindle and piezo positioners to the detector was adjusted until the heat map of the raster scan matched the shape of the crystal (Fig. 3 ▸ *a*). After the initial calibration, the synchronization worked for all frame rates and scanning speeds.

Unlike serial femtosecond X-ray crystallography at FELs, which takes thousands of ‘still’ diffraction patterns from crystals, the sample in our experiment is rotated during raster scan data collection. As a result of the moment of inertia of the rotary stage, the sample is rotated continuously in one direction to synchronize the rotation with the high-speed raster scanning. For all raster experiments, we used proteinase K microcrystals with average sizes of 5–10 µm.

We developed protocols for the two main-use cases of raster scanning, namely ‘rastering for crystal location’ and ‘rastering for data collection’.

In ‘rastering for crystal location’, the aim is to scan the ROI and locate all micrometre-sized crystals. A raster scan with a 2 µm step and 2.5 ms exposure time per frame scans an area of 80 µm × 40 µm in 2 s (Fig. 3 ▸ *b*). Instead of still exposures, the scan was conducted in a ‘nearly still’ way with a rotation of 0.002° per frame to minimize the position mismatch induced by the rotation while retaining the rotation axis as the experiment synchronization master; 10% of the maximum flux resulted in an average dose of 30 kGy. The resultant heat map (Fig. 3 ▸ *b* inset) shows the position of each crystal.

‘Rastering for data collection’ is optimized to achieve the highest data collection speed; therefore, the heat-map function is disabled in this type of raster scan. Various combinations of parameters including raster area, step size and oscillation width were characterized in a set of initial experiments. Given the size of the protein crystals, a 1 µm step size and 0.2° oscillation width were determined empirically to be the optimum settings to enable indexing of the partial datasets (*i.e.* diffraction data from a 1° wedge would be collected by scanning across a 5 µm-wide crystal). Multiple raster scans were performed sequentially over the ROI. The total rotation during a data collection raster scan, as dictated by the number of steps and the oscillation width per step, was limited in the −45° to +45° angular range around the face-on view to minimize multiple crystal exposures. In the current experiments, each raster section was entered separately in the data collection GUI; in the future, a large millimetre-scale raster scan will be automatically partitioned to sub-rasters and performed by combining the fine and coarse scanning stages. Different detector frame rates from 200 Hz to 750 Hz, corresponding to exposure times from 5 ms to 1.334 ms, were tested. Detailed settings of these raster scans are listed in Table S2. The beam attenuation was adjusted based on the exposure time in order to maintain a constant dose (0.30 MGy calculated by *RADDOSE-3D*). At the turning points of the raster lines, the dose deposited on the crystals is higher because of the slower goniometer translation. The border pixels were not evaluated in the data processing.

### 3.3. Data processing   

The raster data from multiple microcrystals were processed using a Python scripted workflow, which employs *DOZOR* (Svensson *et al.*, 2015 ▸), *XDS* (Kabsch, 2010 ▸), *POINTLESS* and *AIMLESS* (Winn *et al.*, 2011 ▸). *XDS* was chosen for its robust handling of poor quality images, such as poor spot shape and weak diffraction, and for its capability to run highly parallelized on our compute cluster. The command line input and output streams of *DOZOR* and *XDS* greatly facilitate their integration with Python.

Diffraction patterns acquired from multiple raster scans were first processed by *DOZOR*, which estimated the diffraction signal of each frame and assigned a scalar figure of merit (Svensson *et al.*, 2015 ▸). Based on *DOZOR*’s figures of merit, the data were divided into multiple partial datasets, with a typical partial dataset containing diffraction patterns from a 1–2° wedge. Each partial dataset was then separately indexed and integrated in *XDS* to determine the space group and unit-cell dimensions from each indexable dataset. Inspired by the *Nearest-cell* PDB database search tool (Ramraj *et al.*, 2012 ▸), the unit-cell size of each dataset was transformed from crystallographic coordinates (*a*, *b*, *c*, α, β, γ) into the default orthogonal system (Bernstein *et al.*, 1977 ▸; Callaway *et al.*, 1992 ▸) to better visualize the indexed partial datasets and remove outliers. The resultant diagonal vectors of the unit cell were displayed in a three-dimensional plot (Fig. 3 ▸ *c*) and were clustered using the *K*-means clustering algorithm (Lloyd, 1982 ▸). Each cluster was processed separately in the subsequent processing. The outliers, as determined by their distance from the center of the cluster, were excluded from subsequent processing.

The remaining datasets were combined *via* *XSCALE* without merging and assuming the space group is *P*1. The result was evaluated by *POINTLESS* to determine the best-fitted space group and the data merged again by *XSCALE* utilizing the given space group. The *SciPy* functions *Linkage* and *Dendrogram* (Jones *et al.*, 2001 ▸) were utilized to perform the hierarchical clustering of the correlation coefficients from *XSCALE*, using the distance definition described by Giordano *et al.* (2012 ▸) and Santoni *et al.* (2017 ▸). The resultant dendrogram was plotted *via* Python *matplotlib* (Hunter, 2007 ▸) for further outlier removal (Fig. 3 ▸ *d*). This two-step outlier rejection, *i.e.* unit-cell clustering followed by correlation coefficient clustering, was found to be necessary as the correlation coefficient clustering was not able to completely distinguish the difference in unit cells. Compared with other unit-cell clustering methods that use the edge lengths (Santoni *et al.*, 2017 ▸) or face diagonal lengths (Giordano *et al.*, 2012 ▸), this coordinate transformation method considers both the length and orientation of the volume diagonal vector, and worked very well for the datasets presented.

After outlier removal, the data merged by *AIMLESS* were used for structure refinement, which was manually performed in *PHENIX*. PDB entry 2id8 was selected as the starting model for *E. album* proteinase K. *COOT* (Emsley *et al.*, 2010 ▸) was used for viewing the electron density maps, adding ligands and adjusting the solvent. The refined structures were validated using the program *PROCHECK* (Laskowski *et al.*, 1993 ▸).

As shown in Table 1 ▸, data of proteinase K with a 200 Hz, 500 Hz and 750 Hz detector frame rate were processed. A total of 30 raster scans were collected at each frame rate, resulting in 391, 385 and 499 partial datasets, respectively. Among those partial datasets, 279, 236 and 316 were found to be indexable, giving an average indexing rate of 65%. After removing those with incorrect unit-cell sizes, a hierarchical cluster-analysis dendrogram with a cutoff limit at a distance of 0.60 was utilized for the final outlier removal. The final datasets, merged from 225, 214 and 299 partial datasets, respectively, were processed to a resolution of 2 Å, limited by the detector distance and area in ROI mode. The corresponding 〈*I*/σ(*I*)〉 in the highest-resolution shell was 3.2 ± 0.2. The overall CC(1/2) was 95.9%, 93.8% and 95.5%, respectively, with 83.6%, 78.5% and 81.8% in the highest-resolution shell. The structure refinement also showed similar *R*_free_ and *B* factors.

At three data collection speeds, both the X-ray diffraction data and the structure refinement demonstrate similar statistics, indicating equal data quality. The data taken at a 750 Hz detector frame rate were collected with 100% transmission using the full flux of the FMX beamline. While scanning the 30 µm × 15 µm area with a 1 µm step, the piezo scanner achieved a 12.5 Hz scanning frequency, surpassing our regular goniometer by more than one order of magnitude. This is currently still limited by the beamline flux, which will be further increased with the NSLS-II ring current from 350 to 500 mA, and by higher dose rates achievable at longer wavelengths.

## 4. Conclusions   

We have developed the FastForward Goniometer, a high-speed, high-precision, piezo-based goniometer system to perform serial microcrystallography using the full flux of the fully focused FMX beamline. The laboratory characterization demonstrated that the system we developed is capable of achieving greater than 40 Hz scanning frequency over a 200 µm scanning range in all three directions with 300 nm repeatability. For scanning frequencies below 5 Hz, the bidirectional repeatability was below 25 nm. The performance of the goniometer system was illustrated by conducting high-speed raster-scanning serial microcrystallography with the detector frame rates varying between 200 Hz and 750 Hz. The resultant protein structures, which were solved *via* a Python scripted automated data-processing workflow, indicated equally high data quality, proving reliable performance of the piezo-scanner at various scanning frequencies. This unprecedented experimental speed significantly reduces serial crystallography data collection time at synchrotrons, allowing utilization of the full brightness of the emerging synchrotron radiation facilities.

## Supplementary Material

Supplementary materials suggested by the reviewers: Figure S1. Scaling factors of the vector scan data. Figure S2. Wilson plots. Table S1. Free R-factors in resolution bins for 200, 500, and 750 Hz frame rate proteinase K datasets. Table S2. Parameters of raster scans. . DOI: 10.1107/S1600577518010354/yn5033sup1.pdf

## Figures and Tables

**Table 1:** X-ray data-collection and refinement statistics for crystallography experiments performed using a piezo scanner at the FMX beamline

	Trypsin	Proteinase K		
Data collection				
Data collection protocol	Vector	Raster		
Detector frame rate (Hz)	20	200	500	750
Piezo scanning speed (µm s^−1^)	1	200	500	750
Piezo rastering frequency (Hz)	–	3.33	8.33	12.5
Partial datasets				
Total	–	391	385	499
Indexed	–	279	236	316
Merged	–	225	214	299
Space group	*P*2_1_2_1_2_1_	*P*4_3_2_1_2		
Unit-cell parameters	*a* = 53.90, *b* = 57.86, *c* = 66.27	*a* = *b* = 68.20, *c* = 107.43	*a* = *b* = 68.20, *c* = 107.06	*a* = *b* = 67.68, *c* = 106.32
Resolution range (Å)	29.20–1.52 (1.56–1.52)	19.63–1.99 (2.04–1.99)	19.57–2.00 (2.05–2.00)	19.43–2.00 (2.05–2.00)
No. of unique reflections	32102	17938	17793	17430
Completeness (%)	96.9 (95.0)	99.6 (96.1)	99.7 (97.1)	99.8 (98.9)
*R*_merge_	0.041 (0.616)	0.282 (0.529)	0.340 (0.579)	0.299 (0.651)
*R*_pim_	0.027 (0.397)	0.118 (0.227)	0.145 (0.251)	0.123 (0.270)
〈*I*/σ(*I*)〉	14.2 (1.6)	5.7 (3.4)	4.5 (3.0)	5.1 (3.0)
CC_1/2_	0.999 (0.677)	0.959 (0.836)	0.938 (0.785)	0.955 (0.818)
Multiplicity	3.0 (3.0)	20.4 (16.3)	20.3 (16.5)	28.2 (22.8)

Refinement				
Resolution range (Å)	29.20–1.52	19.63–1.99	19.57–2.00	19.43–2.00
No. of reflections used in refinement	30803	17053	17347	16858
No. of reflections used for *R*_free_	1766	1712	1726	1679
*R*_work_/*R*_free_	0.178/0.189	0.183/0.216	0.191/0.220	0.186/0.214
No. of atoms				
Protein	1628	2032	2032	2032
Metal ion (Ca)	1	2	2	2
Solvent	164	209	211	212
Ligand (1 NO_3_)	–	4	4	4
*B* factors (Å^2^) of protein	21.3	14.1	11.8	14.5
R.m.s. deviations				
Bond lengths (Å)	0.082	0.005	0.006	0.006
Bond angles (°)	2.479	0.778	0.756	0.779
Average r.m.s. *B* factor	6.15	5.62	5.52	5.25
Ramachandran plot (%)				
Most favored	88.7	89.4	90.2	90.6
Allowed	11.3	10.6	9.8	9.4
Disallowed	0.0	0.0	0.0	0.0
