# Supplementary material for: High-speed raster-scanning synchrotron serial microcrystallography with a high-precision piezo-scanner
Source: J Synchrotron Radiat. 2018 Aug 23;25(Pt 5):1362–70. doi: 10.1107/S1600577518010354 (PMC6140394; doi:10.1107/S1600577518010354)
Supplement: Supplementary file 1 [file s-25-01362-sup1.pdf]

JOURNAL OF
SYNCHROTRON
RADIATION

Volume 25 (2018)

Supporting information for article:

High-speed raster-scanning synchrotron serial micro-crystallography with a high-precision piezo-scanner

Yuan Gao, Weihe Xu, Wuxian Shi, Alexei Soares, Jean Jakoncic, Stuart Myers, Bruno Martins, John Skinner, Qun Liu, Herbert Bernstein, Sean McSweeney, Evgeny Nazaretski and Martin R. Fuchs

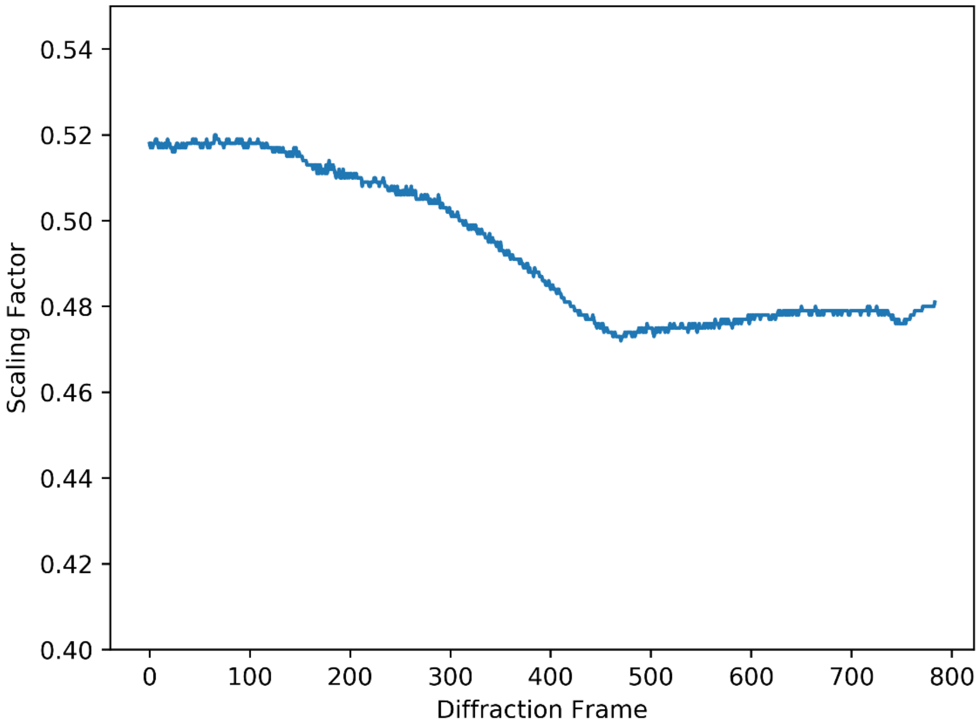

Figure S1. Scaling factors of the vector scan data. The first 800 frames from the vector scan were processed. The scaling factor varies from 0.47 to 0.52.

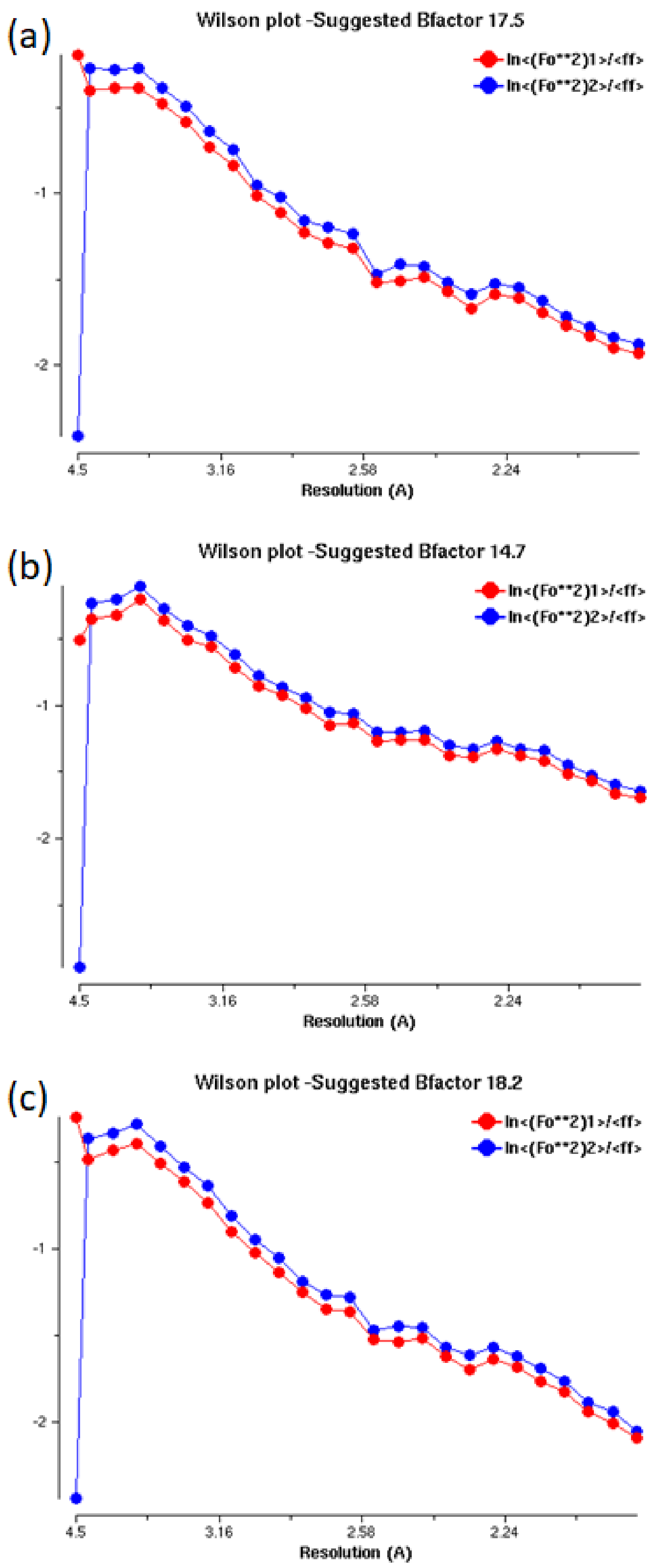

Figure S2. Wilson plots. Plots of (a) 200 Hz, (b) 500 Hz, and (c) 750 Hz framerate proteinase K datasets, generated by WILSON and LOGGRAPH of CCP4.

Table S1. Free R-factors in resolution bins for 200, 500, and 750 Hz frame rate proteinase K datasets.

200 Hz		500 Hz		750 Hz	
Resolution bin	R_{free}	Resolution bin	R_{free}	Resolution bin	R_{free}
19.41 - 4.55	0.209	19.57 - 4.55	0.241	19.43 - 4.55	0.210
4.55 - 3.62	0.185	4.55 - 3.62	0.172	4.55 - 3.62	0.179
3.62 - 3.17	0.223	3.62 - 3.17	0.205	3.62 - 3.17	0.190
3.17 - 2.88	0.191	3.17 - 2.88	0.209	3.17 - 2.88	0.212
2.88 - 2.67	0.208	2.88 - 2.67	0.275	2.88 - 2.67	0.233
2.67 - 2.51	0.233	2.67 - 2.51	0.232	2.67 - 2.51	0.269
2.51 - 2.39	0.230	2.51 - 2.39	0.225	2.51 - 2.39	0.222
2.39 - 2.28	0.229	2.39 - 2.28	0.232	2.39 - 2.28	0.213
2.28 - 2.19	0.234	2.28 - 2.20	0.242	2.28 - 2.20	0.233
2.19 - 2.12	0.203	2.20 - 2.12	0.225	2.20 - 2.12	0.230
2.12 - 2.05	0.232	2.12 - 2.05	0.211	2.12 - 2.05	0.238
2.05 - 1.99	0.315	2.05 - 1.99	0.250	2.05 - 1.99	0.217

Table S2. Parameters of raster scans.

	For crystal location	For data collection		
Detector frame rate (Hz)	400	200	500	750
Number of raster scans	-	30	30	30
Raster area (μm^2)	80 × 40	30 × 15		
Raster scan step size (μm)	2	1		
Exposure per frame (ms)	2.5	5	2	1.334
Exposure time per raster (s)	2	2.25	0.9	0.6
Oscillation width per frame	0.002°	0.2°		
Total rotation from face-on view	0° ~ 6.4°	-45° ~ 45°		
Beam energy (keV)	12.66			
Beam flux (10^{12}ph/s)	0.088	0.65	1.67	1.84
